# Temporal and Spatial Variations of Drought in China: Reconstructed from Historical Memorials Archives during 1689-1911

**DOI:** 10.1371/journal.pone.0148072

**Published:** 2016-02-02

**Authors:** Jinhong Wan, Denghua Yan, Guobin Fu, Lu Hao, Yaojie Yue, Ruoxi Li, Yunpeng Li, Jiangang Liu, Jun Deng

**Affiliations:** 1 China Institute of Water Resources and Hydropower Research, Beijing, China; 2 State Key Laboratory of Simulation and Regulation of Water Cycle in River Basin, Beijing, China; 3 School of Geography, Beijing Normal University, Beijing, China; 4 CSIRO Land and Water Flagship, Private Bag 5, Wembley, Western Australia, Australia; 5 International Center for Ecology, Meteorology and Environment (IceMe), Jiangsu Key Laboratory of Agricultural Meteorology, Nanjing University of Information Science and Technology, Nanjing, Jiangsu Province, China; Beijing Normal University, CHINA

## Abstract

In China, *Zou Zhe* (Memorials to the Throne, or Palace Memorials), an official communication to the emperors of China by local officials, offers an opportunity to reconstruct the spatial-temporal distributions of droughts at a high-resolution. A 223-year, 1689–1911, time series of drought events was reconstructed in this study based on 2494 pieces of *Zou Zhe*. The results show that: 1) on the temporal scale, the drought affected areas, i.e., number of affected counties, showed three peak periods during the last 223 years and nine extreme drought years with more than 300 counties affected have been identified; 2) on the spatial scale, there existed three drought-prone areas in China, i.e., Gansu province and Ningxia Hui Autonomous Region in Northwest China, Shandong, Hebei, and Henan provinces and Tianjin in the North China, and Anhui and Jiangsu provinces in Jianghuai area, respectively; 3) the drought-prone areas have been expanding from North China to South China since the second half of 19^th^ century; 4) on the seasonal scale, summer witnessed the largest number of drought events. Meanwhile, the uncertainties of the results were also discussed, i.e. what caused the spatial-temporal distribution of drought. The results of this study can be used to mitigate the adverse effects of extreme weather events on food increasing and stable production.

## Introduction

Drought strike occurs frequently in china. According to historical records, from 206BC to 1949AD, there had been 1056 drought events [1]. This means at least one drought event took place every two years. In the past 60 years, drought has affected more than 29 million people every year[2]. And it has become an important restricting factor for economic and social development. The economic losses caused by drought accounted for over 15% of the loss from all natural disasters[3]. According to the data from the released *Bulletin of Flood and Drought Disaster in China*, during the past 60 years, 21.56 million hm^2^ of farmland was affected by drought each year, and the annual loss of crop yield caused by drought reached 16.1 billion kg, accounting for over 60% of the total grain loss caused by natural disasters [2]. Under the context of global warming and climate change, instrumental observations indicate that the North China has become warmer and drier over the last four decades, and the annual precipitation has declined by about 43.9 mm [4]. And a further research suggests that the persistent drought in North China won’t effectively alleviate within the next 10 years, and the seasonal drought of South China will become increasingly severe[5]. Coupled with the rapid economic development and population growth, water shortages will become increasingly serious and drought study will become an important issue for China's sustainable development.

Historical archives data are valuable sources of information about past climate [6–7]. Chinese historical documents provide a tremendous amount of valuable data on past weather and climate that can be used to reconstruct the climate over hundreds and perhaps thousands of years[8]. Previous studies have provided extensive and detailed evaluations of Chinese historical documentary data sources with regard to weather and climate phenomena [9–10]. In recent years, there are many Chinese researchers who have engaged in research into spatial-temporal changes of drought. For instance, Wang et al. [11] map temporal and spatial distribution of drought over the past 50 years in China, using the provincial newspapers and disaster reports since 1949. Zhang [12] notes that historical documents specify the locations where and the time when climate changes occur more accurately than tree rings, ice cores and sediments. Ge et al. [8] use the *Yuxuefencun* (rain infiltration depth and snow depth) records during the Qing Dynasty to reconstruct high-resolution, quantitative precipitation data. Zheng et al. [13], using historical disaster documents, have reconstructed the drought temporal-spatial changes in the North China Plain and Jianghuai Plain over the past 1500 years. Shen et al. [14] use the Chinese drought/flood proxy data of the past five hundred years to identify the cases of exceptional drought events in eastern China (east of 105°E), and to study their spatial patterns and temporal evolutions. Liu and Yang[15] analyze the temporal-spatial patterns of the frequency of major natural disasters during the period between 180 BC and 1949AD through the collation of historical documents, reaching to the conclusions that there is a significant relationship between the type of disaster and the spatial distribution of hazard frequency, and the provincial statistics show that in historical period the major droughts mainly occurred in the northern China.

Despite, currently, the research of drought distribution and its changes over the historical periods in China is based on local chronicles, history books, observations and disaster chronology etc., but those historical data have low temporal resolution, and the reliability of data needs to be further discussed. This paper is the first to study temporal and spatial change of drought events from 1689 to 1911 based on the drought disaster data extracted from *Zou Zhe* (Memorials to the Throne, or Palace Memorials), an official communication to the emperors of China by local officials in ancient China. Sorting out information on drought from these historical Memorials archives with county as the basic statistical unit, this paper has reconstructed the temporal and spatial patterns of drought distribution in the period from 1689 to1911 in China. This study provided important validation data for historic drought researches, e.g., those studies based on tree ring, ice core, and has important practical significance for China to optimize agricultural production patterns, to mitigate the adverse impact of extreme weather events on grain yield increase and stabilization, and seeking effective defense measures for sustainable agricultural production and food security.

## Drought Archives Description

This research sorts out the drought hazard data from Memorials archives. The data consist of two parts. Part one, covering the period from 1662 to 1735, is extracted from Memorials archives publications, such as *Memorials to the Throne in the Reign of Emperor Kangxi*, *Emperor Kangxi's Comments in Chinese on Memorials Submitted to Him*, and *the full translations of Emperor Kangxi's Comments in Manchu on Memorials Submitted to Him*, et al. Another part, covering the period from 1736 to 1911, is extracted from the copied files conserved by Water History Department of China Institute of Water Resources and Hydropower Research. Due to wars and local separatist movements etc. the size of drought hazard data in early Qing Dynasty (1662–1688) is neglected; therefore, data collection of this paper actually starts from the year of 1689. In addition, the Taiping Rebellion broke out in 1851 and lasted till 1865, making provinces along the Yangtze River war zones and thus reducing the size of drought data of this area over that period. However, according to Ge (2011), that region was relatively humid at that time.

**Fig 1:**
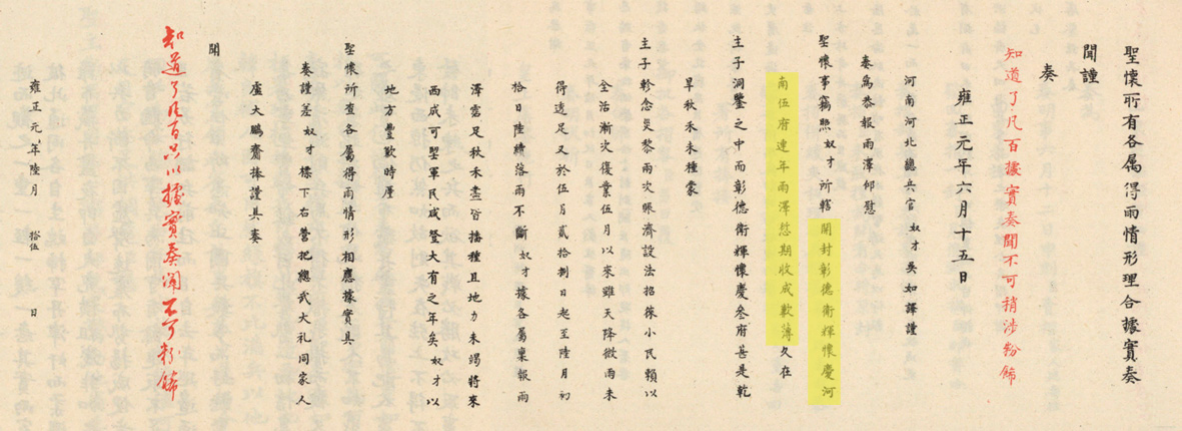
A piece of Memorials in Yongzheng Period (1723-1735AD) in Qing Dynasty. Which contains a record of drought-strike event in AD1723 (the First year of Yongzheng Period). Due to rain delay, at least five different location occurred drought, they are Kaifeng Fu, Zhangde Fu, Weihui Fu, Huaiqing Fu, Henan Fu, respectively (yellow section). Red character is the comment of Emperor Yongzheng.

The selected Memorials archives (Fig 1) should include information such as precipitation shortage, disaster loss or crop failure and post disaster relief. The descriptions of precipitation shortage include "delay of rain", "farmers craved for rain", "long drought", and "the spring and summer droughts", etc. The descriptions of disaster loss or crop failure are “grain withered", "the loss of revenue due to drought", "a scene of utter desolation where not even a blade of grass grew", "the hungry were forced to feed themselves with tree bark and grass root", "people sold large pieces of land only for food that could support the family for one month", "hunger hung everywhere", etc. And post disaster relief measures include pension, tax exemption, soup kitchens, and "food for work" project.

The central government of Qing dynasty attached great importance to disaster data collection, and established a disaster information reporting system. The provinces principal officials must report disaster information to the central government regularly. At the same time, The Qing government also established a multiple source disaster verification system, which ensured the authenticity and reliability of the information on Memorials [16]. In addition, it needs to be pointed out that disaster information on Memorials archives is more authoritative and credible than and can be verified by local chronicles, private notes and other historical data.

Table 1 shows that out of 223 years (1689–1911), 181 years witnessed the occurrence of drought and 2494 pieces of Memorials which record drought events are collected. The average number of Memorials mentioning drought collected for each year is 13.78, and the period of the reign of Emperor Guangxu saw the largest average number of memorials collected for each year is 28.12; the period during which Emperor Jiaqing reigned ranks the second with an average of 17.64 memorials per year; presumably, the reign of Emperor Kangxi saw the smallest frequency of drought, with only 2.24 memorials mentioning that per year.

**Table 1:** Number of drought archives in different periods of Qing Dynasty (1689–1911).

Periods	Kangxi	Yongzheng	Qianlong	Jiaqing	Daoguang	Xianfeng	Tongzhi	Guangxu	Xuantong	Total
years have archives	17	9	49	22	28	10	10	33	3	181
Numbers of archives pieces	38	62	601	388	332	52	52	928	41	2494
average	2.24	6.89	12.27	17.64	11.86	5.20	5.20	28.12	13.67	13.78

**Fig 2:**
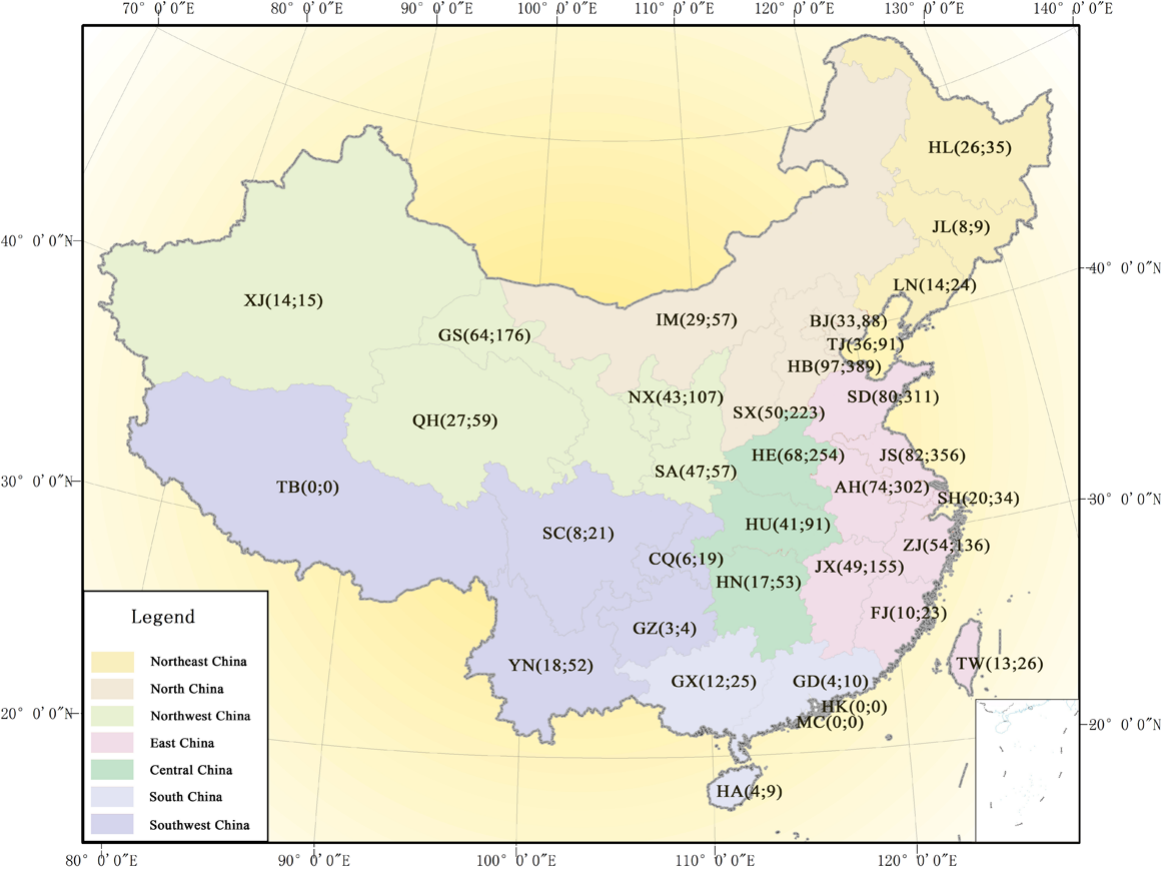
The drought Memorials archives distribution at provincial level. The HL (26; 35) stands for Heilongjiang province have suffered 26-years drought strikes during 1689–1911, and exists 35 pieces of drought Memorials. There divide seven administrative zones in China, these are (1) the Northeast China which covering Heilongjiang (HL), Jilin (JL), and Liaoning; (2) the North China which covering Beijing (BJ), Tianjin (TJ), Hebei (HE), Shanxi (SX), and Inner Mongolia; (3) the Northwest China covering Shaanxi (SA), Ningxia (NX), Gansu (GS), Qinghai (QH), and Xinjiang (XJ); (4) the East China which covering Shandong (SD), Jiangsu (JS), Anhui (AH), Shanghai (SH), Zhejiang (ZJ), Jiangxi (JX), Fujian (FJ), and Taiwan (TW); (5) the Central China which covering Henan (HE), Hubei (HU), and Hunan (HN); (6) the South China which covering Guangxi (GX), Guangdong (GD), Hainan (HA), Hong Kong (HK), and Macau (MC); and (7) the Southwest China which covering Chongqing (CQ), Guizhou (GZ), Sichuan (SC), Yunnan (YN), and Tibet (TB), respectively.

The Fig 2 indicates that North China, Central China, East China, and Northwest China have more drought Memorials archives than other regions from 1689 to1911, especially Hebei, Jiangsu, Shandong, Anhui, Henan, Gansu, Shanxi, Jiangxi and Shaanxi provinces. For example, there are 389 pieces of Memorials reporting drought in Heibei province in the 97 years from 1689 to 1911, making it 4 pieces per year on average. Meantime, Tibet autonomous region, Hong Kong and Macau submitted no Memorials mentioning drought.

## Result and Discussion

### Statistical characteristics of drought archives

According to memorials, from 1689 to1911, the average annual number of affected counties was 70. In 56 years, droughts affected more than 100 counties every year; in 19 years, over 200 counties were influenced; and in 9 years, more than 300 counties were impacted. For instance, the numbers of affected counties were as large as 602 in 1877 AD, 410 in 1785 AD, and 408 in 1878 AD. Table 2 reflects the time distribution of droughts that affected more than 100 counties (1689–1911).

**Table 2:** Drought years in different affected areas.

Threshold	number	year
affected areas are 100∼199 counties	37	1723, 1737, 1738, 1744, 1774, 1794, 1802, 1803, 1807, 1810, 1812, 1817, 1818, 1820, 1825, 1826, 1829, 1832, 1836, 1838, 1839, 1840, 1846, 1847, 1875, 1879, 1880, 1882, 1884, 1885, 1886, 1887, 1889, 1890, 1894, 1898, 1901.
affected areas are 200∼299 counties	10	1745, 1792, 1811, 1813, 1814, 1837, 1873, 1891, 1893, 1899.
affected areas are more than 300 counties	9	1759, 1785, 1835, 1876, 1877, 1878, 1888, 1892, 1900.

### Temporal characteristics of drought events in Qing Dynasty

Because of the historical archives on disaster often record “Abnormal or Calamity” not "Normal", historical drought archives were often discontinuities in time scale and spatial scale. Previous studies [16] pointed out that if a regional’s disaster archives were interrupted within three years, the climate in this period could be considered normal or no disaster in this region. In other words, there are no adverse climate or weather events such as drought, flood, etc. If archives was interrupted more than three years, it could be considered missing data of disaster in specific region. There might be drought or flood events. According to the principle above, the drought memorials archives recorded from 1689 to 1911 was a continuous time series.

**Fig 3:**
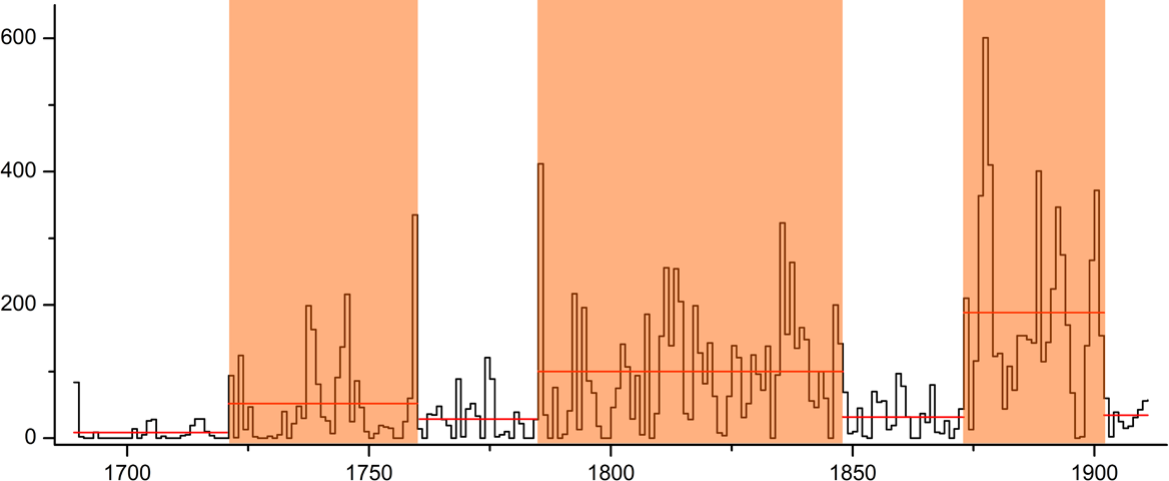
Annual number of drought strikes counties recorded in the historical Memorial archives during the 223-year period from 1689 to 1911. Red line is the average number of above mentioned periods. It is obviously the numbers of drought affected counties show an increasing trend. The red section is three peak periods of drought.

During the 223 years, 181 years witnessed drought strikes according to the records. This translates into 8.1 drought-stricken years per decade on average. Fig 3 shows a year-by-year compilation of drought-struck counties between 1689 and 1911. According to this figure, the annual number of counties struck by drought is on the rise, and there were three peak periods of drought (Fig 3). The first one lasted 38 years from 1722 to 1759, and the average number of drought affected countries is 52 per year. The second lasted 63 years, the average number rose to 100 per year. The third is from 1873 to 1901and the average number is 188 per year. In five years, the annual numbers of affected counties are above 300, especially the period between 1876 and 1878 when drought hit three years in a row. This result is consistent with the previous study conducted by National Research Group of Major Natural Disaster of China [17], and it needs to be pointed out, this study has much better spatial resolution compared with NRGMND’s research.

### Spatial distribution in Qing Dynasty

**Fig 4:**
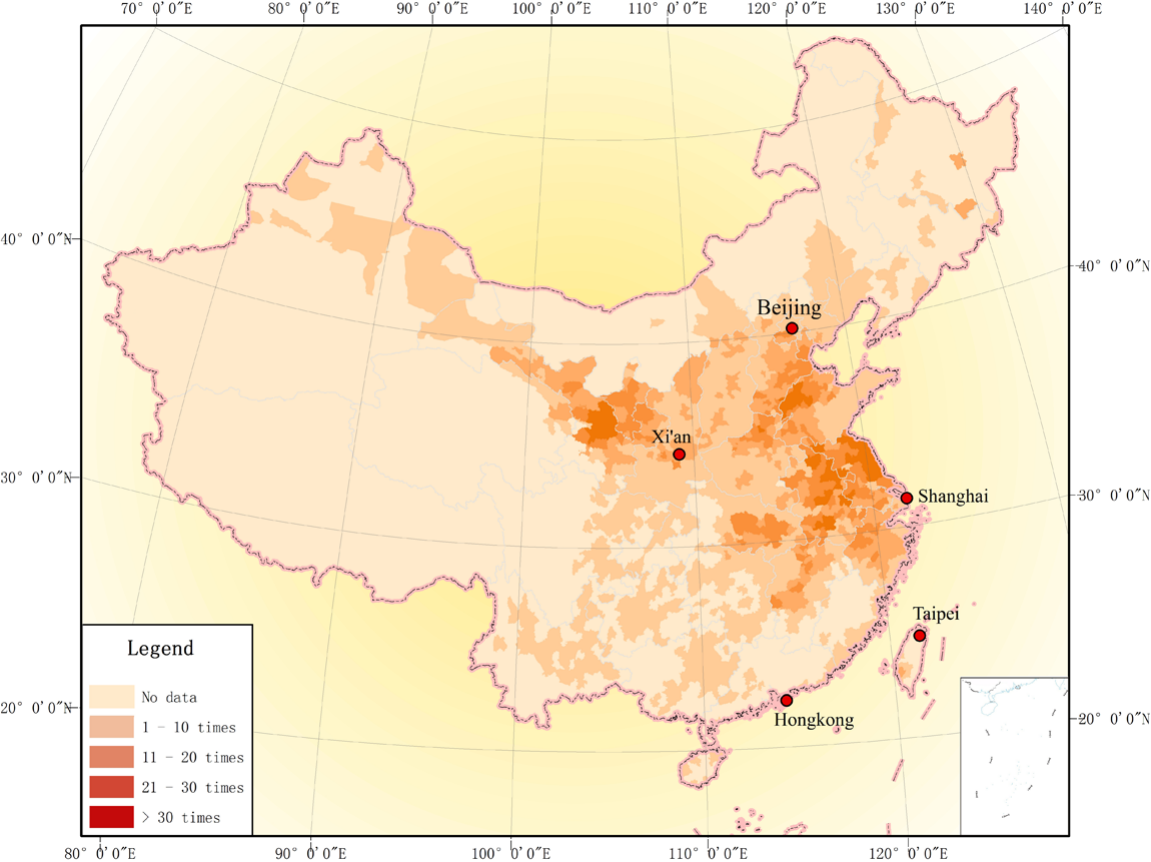
Drought frequency distribution of counties in Qing Dynasty (1689–1911).

The Map of Drought Distribution in Qing Dynasty (Fig 4) indicates that the drought spatial distribution characteristic during 1689–1911 is scattered on the whole and concentration in certain small areas. Being scattered on the whole means that nearly all provinces of China have drought records. Concentration in certain small areas means that, while drought was wide-spread in China, there were three drought-prone areas. They were the drought-prone area of Northwest China, covering Gansu, Ningxia and Shaanxi provinces; the drought-prone area of Haihe River Basin, covering Beijing, Tianjin, Hebei, Shanxi, Shandong and Henan provinces; and the drought-prone area of Huaihe River—Yangtze River region, covering Anhui, Jiangsu, Hubei, Zhejiang and Jiangxi provinces.

Not all regions have complete memorial archives on drought. Regions lagging behind in terms of political, economic, social and agricultural development, such as Qinghai, Tibet, Xinjiang and the Northeast China, lack such records. The reasons for this are mainly due to:

1. In early Qing dynasty, Qinghai-Tibet region (including the western part of Sichuan province) was dominated by *Khoshut and Rutter Mongolians Department*. After the war, the Qing government established an autonomous government in this region in mid-18th century. Thus, the little drought information within the existing Memorials archives is limited to the northeastern part of Qinghai province.
2. Wars continued in Xinjiang region (including western Inner Mongolia) in early Qing dynasty. Xinjiang region was not fully vested by Qing government until 1755. Then, the Qing government organized settlements in the Tianshan Mountain area. In 1887 AD, the total area of cultivated land in Xinjiang expanded to 0.77 million ha[18]. The droughts recorded in the existing archives mainly occurred in the oasis of Tianshan Mountain area and the Ili River basin.
3. In Northeast China (including eastern Inner Mongolia), cultivation was prohibited in the early Qing Dynasty. Only part of the land along the Liaohe River was reclaimed. After the First Opium War (1840 AD), the ban was lifted gradually. With agricultural migrants increasing, *Bodune* (now in Fuyu county, Jilin province), *Ningguta* (now in Ningan county, Heilongjiang province) and *Sanxing* (now in Yilan county, Heilongjiang province) became their major settlements [19]. Large-scale agricultural production began, and so did the recording of droughts in memorials.

In addition, the number of regional disasters files may be affected by “bureaucratization of native officers” policy in Sichuan, Yunnan and Guizhou provinces. However, according to Ge [20], China’s climate was relatively humid in Qing dynasty; therefore disasters on the south side of the Yangtze River recorded in the existing memorial archives are mainly floods, while droughts recorded were concentrated in Hunan, Hubei, Jiangsu and Zhejiang provinces. Thus, the reconstructed drought distribution on the south side of the Yangtze River was in line with the reality of the historical period.

### Spatial-temporal distribution of droughts in different periods

**Fig 5:**
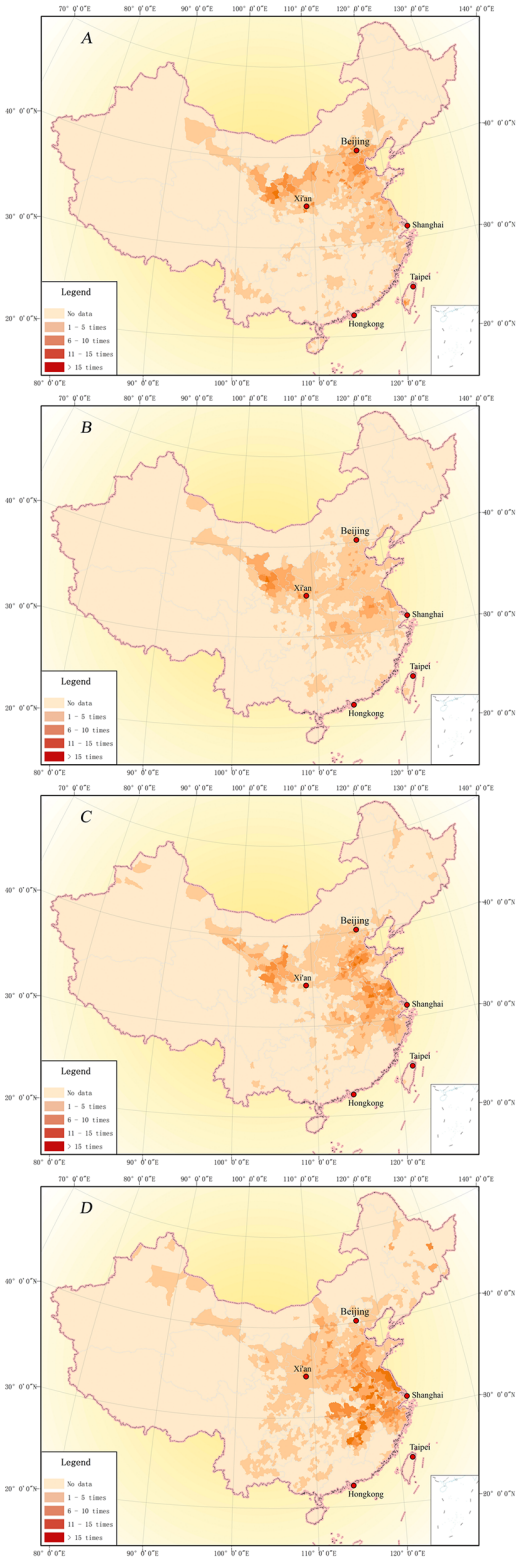
Drought events distribution in different periods. They are 1689-1761(A), 1762-1811(B), 1812–1861 (C), and 1862–1911 (D), respectively.

Investigating the change of drought distribution characteristics is probably the first step of understanding the impact of climate change. In this section, the memorials are categorized into four groups based on the time of submission. As Fig 3 show that the memorials is less in early time of Qing dynasty, so the first period lasted 73years from 1689–1761 (Fig 5A). The rest three periods lasted 50 years, they are 1762-1811(Fig 5B), 1812–1861 (Fig 5C), and 1862–1911 (Fig 5D), respectively. And the Fig 5 shows that the drought prone areas have a bigger change.

In the first period (Fig 5A), drought was more likely to occur in north China, such as Hebei and Shanxi, and Northwest China, such as Ningxia, and Gansu. For instance, the drought records are more than 10 times in Gansu at first period. In the next half century (Fig 5B), the frequency of drought in Northwest China, according to the records, increased significantly, for example, drought stroke more than 15 times in 50 years in Lanzhou, the capital city of Gansu Province. There seemed to be no change in North China. Contrast to first period (1689–1761), sums of drought records increased significantly in the East China and Central China, such as drought records number of Anhui province up to 10 times. From 1812 to 1861 (Fig 5C), the existing drought memorials show a new pattern of distribution with three drought-prone areas which are located at North China, Northwest China, and East China respectively. In the fourth period (1862–1911), according to Fig 5D, the drought-prone areas expanded from north to south, east and central China, and most part of Jiangsu, Anhui, and Jiangxi Provinces suffered drought strikes in more than 15 out of 50 years.

In this section, the drought affected areas have obviously changes in different periods, like Fig 5 show drought prone areas from North China move to South China since the second half of 19^th^ century.

### Spatial-temporal distribution of 1876–1878 drought event

**Fig 6:**
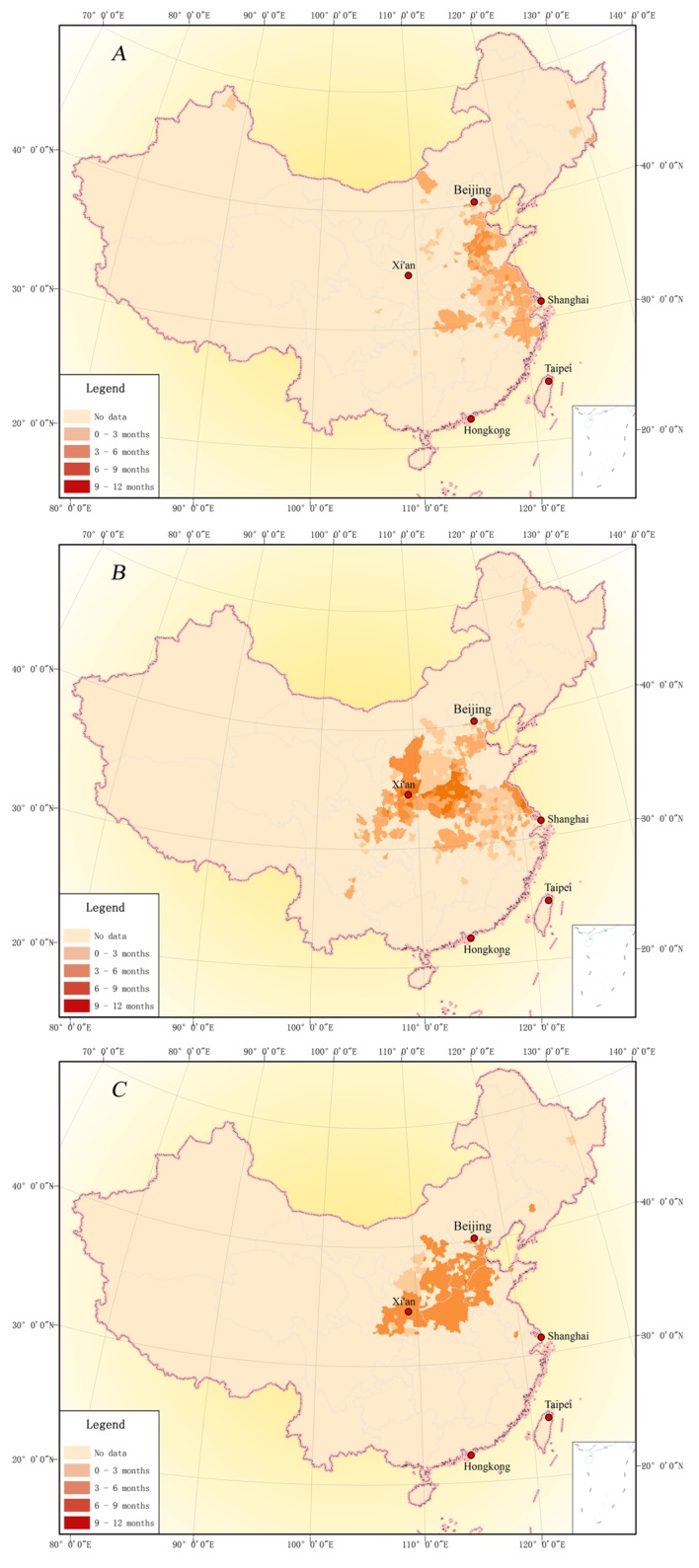
Drought-strikes counties and duration in 1876 (A), 1877 (B), and 1878 (C) of China.

Long-lasting droughts usually result in water shortage and degradation of ecosystem functions [21] and even hinder the progress of human civilization[22–23]. Between 1876 and 1878, a large-scale drought hit China, resulting in one of the driest periods of the past 300 years [12]. This drought started in the spring of 1876 and ended in the autumn of 1878, affecting 11 provinces, i.e., Inner Mongolia, Hebei, Shanxi, Shaanxi, Henan, Shandong, Gansu, Sichuan, Hubei, Anhui and Jiangsu. In this section, the distribution of droughts is mapped (Fig 6) based on information provided by memorials and the spatial-temporal changes of drought-stricken areas are analyzed.

In 1876, a summer drought stroke the northern China. Beijing, Tianjin, Hebei, Shandong, Jiangsu, Anhui, Zhejiang, Hubei, and parts of Shanxi and Inner Mongolia suffered due to little or no rainfall since the spring of 1876 (Table 3). Fig 6A shows that Jiangsu, Zhejiang, and apart of Heibei suffered for more than three months. And the drought ravaged the northwestern part of Shandong province for 9 months. But the situation improved a little from south to north starting from the autumn of 1876.

**Table 3:** Examples of historical records on the 1876–1877 extreme drought episodes.

Region	1876	1877
**Shandong**	“The spring and summer were quite dry and the wheat was in failure harvest. The grain output was even lower than 1 grade. It was difficult to plough and sow at planting season, and a large number of wheat were dead. . .”	
**Zhili (today Hebei,Beijing and Tianjin)**	“The food prices were very expensive in *Shuntian Fu*, due to the drought since the summer and harvest damages. Poor people were very hard to make a living”	“This year was rather dry and locust plagued in some areas”. “Every county of *Shuntian Fu* experienced crop failure due to the dry summer. Expensive prices led poor people to suffer from starvation”
**Henan**		“Drought was awfully extensive this year. The officials had to buy grains from Hubei and Anhui to add into the little left barn”
**Shanxi**	“Taiyuan and other *Fus* were in dry summer, and the autumn harvest was quite poor, especially in *Jiexiu* and *Pingyao* county of *Fenzhou Fu*. The taxation of spring grain should be delayed until the bumper autumn harvest, and people were exempt from all kinds of labor services”	“Long-lasting drought brought very serious disasters”. “Due to the failure autumn harvest in prior year together with persistent drought in this spring and summer, every county opened the official barn to relieve lots of starved people”
**Shaanxi**		“The wheat harvest was very poor due to the delayed spring rainfall. Furthermore, the drought was severe from summer to autumn, and autumn harvest was hopeless

Source: Hao et al.[22].

Compared with 1876, in 1877(Fig 6B), the impact of drought moved northwestwards, and the affected area dwindled in Shandong and Zhejiang, and remained large in Heibei, Shanxi, Henan, Shaanxi, Jiangsu, Anhui, Heibei, and part of Sichuan (Table 3). Lasting longer than that in 1876, the drought in 1877 led to severe aridity. For instance, in the northern part of Henan, central part of Shaanxi, and coast line of Jiangsu, drought lasted longer than 9 months, even reaching 12 months.

From the winter of 1877 to the summer of 1878(Fig 6C), drought lasted nearly 9 months in Tianjin, Hebei, Henan, and apart of Shandong and Shaanxi provinces. As rainfall materialized in autumn of 1878, the three-year drought was finally over.

### Spatial distribution characteristics of seasonal drought

**Fig 7:**
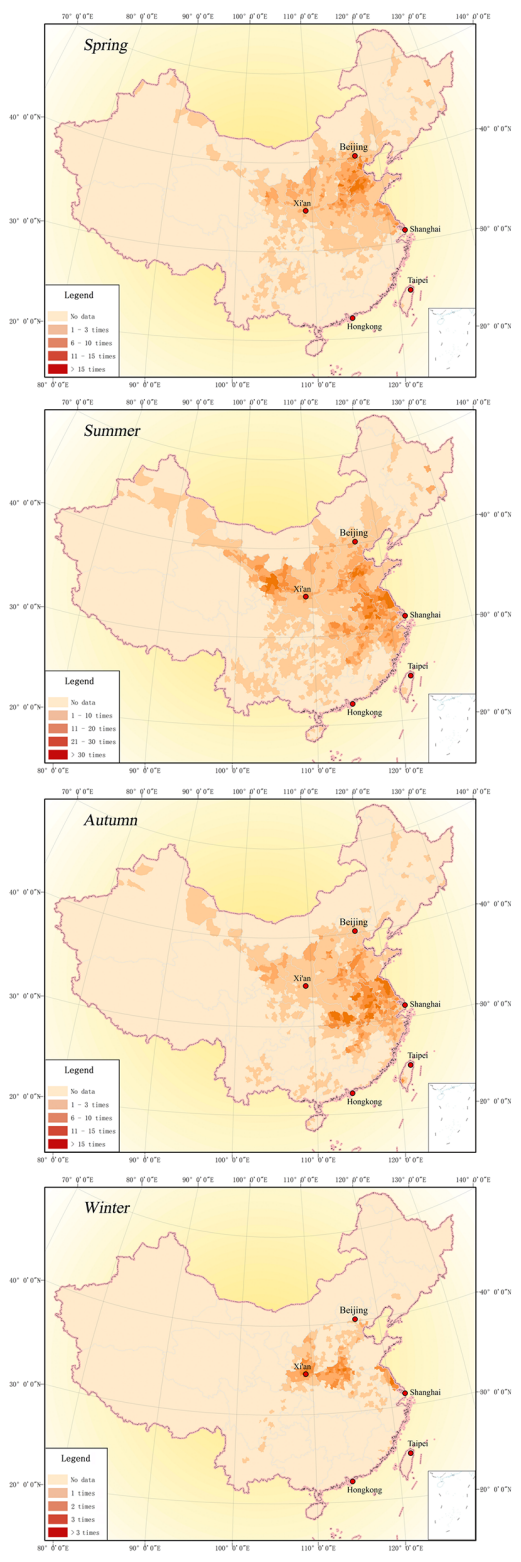
Seasonal drought distribution in Qing Dynasty (1689–1911).

Agriculture is inherently sensitive to drought[24]. Annually, drought causes a large number of crops to fail, especially in crop growth period, seriously hindering agricultural production which is key to ensuring food security and welfare of 1.3 billion people in China [5, 25]. Therefore, a map of spatial distribution of drought in each season is drawn in this section to illustrate the seasonal characteristics of drought. Fig 7 shows drought distribution in different seasons between 1689 and 1911.

Fig 7 indicates that: (1) spring drought took place less than 25 times. The drought-prone area was mainly in Huang-Huai region where Beijing, Tianjin and Hebei were the worst-hit. In most areas, drought occurred once every nine years. (2) In the most extreme/frequent cases, summer drought occurred 46 times. This mainly happened in three areas, namely Shaanxi, Gansu, Ningxia of Northwest China, Beijing, Tianjin and Hebei of the Haihe River Basin, and Jiangsu, Henan, Anhui of the Huaihe River region. In most areas, drought occurred once every five years. In addition, summer drought was also very severe at the downstream of Hanjiang River, Poyang Lake Basin and Taihu Lake Basin. Most of these areas drought occurring were once every nine years. (3) Autumn drought occurred less than 30 times and was mainly at the downstream of the Yangtze River and Huaihe River Basin. The frequency in most areas was once every nine years. In addition, autumn drought was also very serious in the south of the Haihe River Basin and the middle part of the Yellow River region, with a frequency of once every 15 to 20 years. (4) Winter drought occurred less than 4 times and was mainly at the Weihe River basin, the northern part of Henan and the coastal area of Jiangsu province.

The findings above suggest in the agricultural society of ancient China, the central government attaches great importance to the monitoring and control of drought due to its adverse impact on food production. Though with the influence of summer monsoon, the eastern China received more rainfall in summer than in other seasons, an occasional lack of precipitation still raised great concerns among government officials of all levels.

## Conclusion

Memorials on drought in Qing Dynasty were one of the most authoritative, systematic, continuous and reliable historical records of drought from the early 17th to 20th century. This study has referred to 2494 files on drought. These materials are of important scientific value to the study of the spatial-temporal distribution of drought and the social response mechanism of typical drought events from the 17^th^ to the19^th^ century. By reconstructing the temporal and spatial distribution pattern of drought in Qing Dynasty and securing findings that are consistent with historical facts, this study can serve as a reference for future researches on how to mitigate the impact of drought on agricultural production.

1. In terms of temporal distribution of drought, the area affected was on the rise and there were three peaks. If a drought-stricken area as large as 300 counties is the determining condition for being a typical drought-year, there were 9 typical drought-years, namely 1759, 1785, 1835, 1876, 1877, 1878, 1888, 1892, and 1900, of which 1876, 1877, and 1878 are consecutive drought years.
2. As to the spatial distribution, there are mainly three drought-prone areas, namely Gansu and Ningxia in Northwest China, Shandong, Hebei, Henan and Tianjin in North China and Anhui and Jiangsu in Jianghuai Region. In addition, Hubei, Jiangxi, Zhejiang and Taihu Lake Basin were also drought-prone areas.
3. The drought-prone areas have undergone significant changes over time, expanding from North to South China starting from the second half of 19th century.
4. As to the seasonal characteristics of drought, summer drought was the dominant type in Qing Dynasty, affecting Shaanxi, Gansu, Ningxia in Northwest China, Beijing, Tianjin and Hebei in Haihe River Basin, and Jiangsu, Henan and Anhui in Huaihe River basin.

This paper give a new picture of the qualitative meteorological observations available for the early instrumental period, and the potential for climate reconstruction over the past five hundred years especially in China. This study make an important contribution to our understanding of the climatic changes that happened in East Asia during the 17^th^ and 20^th^ centuries.
